# A case for neuroscience in mathematics education

**DOI:** 10.3389/fnhum.2014.00314

**Published:** 2014-05-21

**Authors:** Ana Susac, Sven Braeutigam

**Affiliations:** ^1^Department of Physics, Faculty of Science, University of ZagrebZagreb, Croatia; ^2^Department of Psychiatry, Oxford Centre for Human Brain Activity, University of OxfordOxford, UK

**Keywords:** mathematics, education, learning, problem solving, cognitive development, brain imaging, society

Mathematics lies at the heart of science and technology impacting on the economic performance of societies since ancient times (OECD, 2010). At the level of individuals too, the development of mathematical proficiency appears correlated with individual development and career prospects across a wide range of professions (RAND Mathematics Study Panel and Loewenberg Ball, 2003). It does not come as a surprise to realize that mathematics education traces back several thousand years. However, still very little is known about the fundamental principles of how individuals learn mathematics and at which age education should start. The issue is far from trivial as it is commonly assumed that mathematics is a special subject area perhaps requiring specific motivation, interest and teaching methods in order to be learned efficiently (National Council of Teachers of Mathematics, 2000). Here, we are attempting to make a case for neuroscience methodology as a modern tool capable of contributing to the debate, where a special but not exclusive emphasis is on brain development. Note that for the purpose of this opinion paper, neuroscience is essentially equated with magnetic resonance imaging (MRI), as MRI based approaches currently constitute mainstream research in this field of study according to our understanding.

Developmental studies are increasing our understanding of maturational changes in the human brain (Blakemore, 2012). In particular, structural MRI studies reveal an increase in white matter volume during childhood and adolescence suggesting an increase of connectivity in the developing brain (Giedd and Rapoport, 2010). Interestingly, gray matter volume is characterized by an inverted-U shaped curve peaking at different age in different brain regions (Giedd et al., 1999), which suggests a non-linear, heterogeneous trajectory where proficiencies mature at different times and speeds dependent on which brain regions are most important for a given skill. For example, it is commonly agreed that the intuitive sense of number or quantity is an early ability that can be observed already in infants and that can predict mathematical proficiency later in life (Starr et al., 2013).

In addition to structural studies, functional neuroimaging provides further insight relevant to mathematics education. For example, a developmental functional MRI study of mental arithmetic has shown that the pattern of brain activation changes with student age (Rivera et al., 2005). Importantly, these age-related changes were associated with functional maturation rather than alterations in gray matter density. Moreover, functional studies can help to elucidate the role of specific brain regions in mathematical processing. For example, it has been suggested that the intuitive understanding of quantities is associated with activity in the intra-parietal sulcus (Dehaene, 1997) and, more generally, parietal cortices that are involved in various mathematical tasks from number comparison to complex processing such as proportional and deductive reasoning (e.g., Kroger et al., 2008; Vecchiato et al., 2013). However, additional studies are needed to establish links between development of brain structures and their functional maturation.

Many neuroimaging studies have focused on development of arithmetic skills in children and adults (for a review see Zamarian et al., 2009). Again, different parts of the parietal cortex, such as bilateral intra-parietal sulcus and left angular gyrus, are shown to have a crucial role in mental calculations (e.g., De Smedt et al., 2011; Grabner et al., 2013). In contrast, other brain areas appear to mature relatively late, such as prefrontal association areas thought to be involved in mathematical cognition and other higher-order processes developing throughout childhood and adolescence (Blakemore, 2012). Such insight might shed some light on the transition from concrete arithmetic to the symbolic language of algebra, where students have to develop abstract reasoning skills that enable them to generalize, model, and analyze mathematical equations and theorems (e.g., Qin et al., 2004; Lee et al., 2007; Anderson et al., 2012).

Ultimately, mathematical proficiency will require the coordinated action of many brain regions as exemplified by an influential model of algebraic equation solving (Anderson et al., 2008). Based largely on functional MRI studies of brain activation, the model stipulates distinguishable functional modules that map onto anatomically separate brain regions. For example, a visual module that extracts information about the equation is associated with the fusiform gyrus. An imagery module holding a representation of the equation and performing transformations on the equation is located in posterior parietal cortices. A module responsible for retrieval of previously learned algebraic rules is associated with the left prefrontal cortex. Such models are important as they help to devise methods to track mental states in individuals solving algebraic equations (Anderson et al., 2012). Thus, neuroscience could conceivably help to better understand the relationship between biological brain development and the development of the human capacity for mathematical cognition mediated by educational experience (Royer, 2003).

More specifically, longitudinal studies of changes in brain activation with practice in equation solving (Qin et al., 2004) confirm what educators have known since ancient times—continued exercise in problem solving is very important. This is non-trivial as such studies offer independent insight about the time needed for practice to yield robust effects on brain activity. In principle, such changes in brain activity can be used to compare different teaching methods at the neuronal level. For example, a study investigating the neuronal correlates of algebraic problem solving by two different methods that are taught in schools in Singapore (Lee et al., 2007) suggested that the more symbol oriented a method was, the higher was the load on the attention system of the brain, which might help to explain why symbolic manipulations are usually considered difficult.

In this context, a number of neuroimaging and neuropsychology studies have demonstrated that the relationship between number and space processing is reflected in the organization of parietal circuits assumed to be associated with these skills (Hubbard et al., 2005). Thus, a better understanding of number and space processing in the brain might conceivably yield guidelines informing teachers how to develop both concepts in parallel. Developing skills in parallel might go further than numbers and space, as there is emerging evidence that pattern recognition that is important in algebraic reasoning (Susac et al., 2014) is closely related to visual attention and visual brain regions (Anderson et al., 2008).

Research efforts have also focused on dyscalculia, a specific learning difficulty in understanding numbers and operations with numbers. Mathematics teachers and parents should be aware that the prevalence of developmental dyscalculia is about 5–7% (Butterworth et al., 2011). Only joint effort of mathematics educators and neuroscientists can lead to better understanding of developmental trajectories of dyscalculia and possible positive effects of early diagnosis and interventions. There is growing evidence that insight gained from neuroscience can inform computer-assisted interventions. For example, neuroscience based computer games have been shown to improve the number comparison ability in children with low numeracy skills (Wilson et al., 2006; Räsänen et al., 2009).

In particular, The Number Race is an adaptive software program designed for teaching number sense to young children aged 4–8. It trains children on the entertaining numerical comparison task developing counting and simple arithmetic skills (1-digit addition and subtraction). It is designed to strengthen links between symbolic and non-symbolic representations of number (concrete sets, digits, and number words). Attention and motivation of children is maintained by adjusting the level of task difficulty so that the success rate stays at around 75%. The rewarding environment may help with other problems, which can be associated with dyscalculia such as attention deficit and hyperactivity disorder (ADHD). Moreover, The Number Race and similar computer-assisted interventions can advance mathematics learning and achievement also in typically developing children (Griffin, 2004).

This game is based on current understanding of the neural circuits involved in numerical cognition, in particular the parietal cortices (Dehaene et al., 2003). However, a caveat is in order. A recent review revealed that only 3 out of 20 mathematics intervention software packages reported the use of neuroscience research as a tool in intervention development (Kroeger et al., 2012). Moreover, the majority of programs reviewed (15/20) lacked any empirical validation, preventing teachers from making informed decision on implementation of such programs in the classroom. Evidently, further empirical, peer-reviewed research is needed to evaluate existing software packages and to guide further developments.

There are challenges. From the early days of educational neuroscience, there have been skeptical views on the possibility of direct classroom application of neuroscientific data (as a “bridge too far” in the words of Bruer, 1997). The increasing public visibility of neuroscience has led to what some scholars call neuromyths, i.e., certain beliefs turned into facts because of having been expressed ever so often through virtually all communication channels, such as the view that some people are left-brained and some are right-brained, or that humans use only 10 percent of their brains. Worryingly, unsubstantiated, neuromyth based teaching and learning methods are in use or have been advertised to teachers and education professionals (Goswami, 2006). This reinforces the notion that insight obtained from high-quality neuroscience must be presented in a non-specialists form to mathematics educators, parents, and politicians so that informed decisions on educational issues can be made (building “bridges over troubled waters” in the words of Ansari and Coch, 2006).

In summary, we are inclined to argue that neuroscience can eventually impact on mathematics education by providing hints as to (a) what mathematics curriculum should be provided at which age, (b) which skills should be developed in parallel, and (c) how to reliably assess the effects of early diagnosis and interventions in the case of specific learning disabilities. Research on the timing of maturation of brain areas involved in mathematical cognition appears particularly important as some economic models propose that earlier economic investment in education, i.e., in preschool programs, always lead to larger economic return than later investments (Cunha and Heckman, 2007). There is neuroscientific evidence, however, that indicates continuing development of executive functions throughout childhood and adolescence. Thus, educational policy makers should be aware of the current neuroscience findings when deciding on the timing of educational investment (Howard-Jones et al., 2012).

We believe that neuroscience will not and should not obviate behavioral and psychometric studies that provide independent insight facilitating the development of new experimental paradigms for neuroimaging studies. One should be clear that neuroscience findings have not made it directly into the mathematics classroom at present. However, this should not deter research and we would like to urge investigators not only to continue but also to extend their study of educational neuroscience. Groundbreaking thoughts take time to mature and to find direct applications, as in the case of Carnot's thermal efficiency theorem. As Carnot's work set up a framework for design of more efficient engines that were constructed decades later, neuroscience research today is setting the scene for future developments in mathematics education.

## Conflict of interest statement

The authors declare that the research was conducted in the absence of any commercial or financial relationships that could be construed as a potential conflict of interest.

## References

[B1] Anderson J. R. Betts S. Ferris J. L. Fincham J. M. (2012). Tracking children's mental states while solving algebra equations. Hum. Brain Mapp. 33, 2650–2665 10.1002/hbm.21391 21932262 PMC6870520

[B2] Anderson J. R. Fincham J. M. Qin Y. Stocco A. (2008). A central circuit of the mind. Trends Cogn. Sci. 12, 136–143 10.1016/j.tics.2008.01.006 18329948 PMC5473424

[B3] Ansari D. Coch D. (2006). Bridges over troubled waters: education and cognitive neuroscience. Trends Cogn. Sci. 10, 146–151 10.1016/j.tics.2006.02.007 16530462

[B4] Blakemore S. J. (2012). Imaging brain development: the adolescent brain. Neuroimage 61, 397–406 10.1016/j.neuroimage.2011.11.080 22178817

[B5] Bruer J. T. (1997). Education and the brain: a bridge too far. Educ. Res. 26, 1–13 10.3102/0013189X026008004

[B6] Butterworth B. Varma S. Laurillard D. (2011). Dyscalculia: from brain to education. Science 332, 1049–1053 10.1126/science.1201536 21617068

[B7] Cunha F. Heckman J. (2007). The technology of skill formation. Am. Econ. Rev. 97, 31–47 10.1257/aer.97.2.31

[B8] Dehaene S. (1997). The Number Sense. New York, NY: Oxford University Press

[B9] Dehaene S. Piazza M. Pinel P. Cohen L. (2003). Three parietal circuits for number processing. Cogn. Neuropsychol. 20, 487–506 10.1080/02643290244000239 20957581

[B10] De Smedt B. Holloway I. D. Ansari D. (2011). Effects of problem size and arithmetic operation on brain activation during calculation in children with varying levels of arithmetical fluency. Neuroimage 57, 771–781 10.1016/j.neuroimage.2010.12.037 21182966

[B11] Giedd J. N. Blumenthal J. Jeffries N. O. Castellanos F. X. Liu H. Zijdenbos A. (1999). Brain development during childhood and adolescence: a longitudinal MRI study. Nat. Neurosci. 2, 861–863 10.1038/13158 10491603

[B12] Giedd J. N. Rapoport J. L. (2010). Structural MRI of pediatric brain development: what have we learned and where are we going? Neuron 67, 728–734 10.1016/j.neuron.2010.08.040 20826305 PMC3285464

[B13] Goswami U. (2006). Neuroscience and education: from research to practice? Nat. Rev. Neurosci. 7, 406–411 10.1038/nrn1907 16607400

[B14] Grabner R. H. Ansari D. Koschutnig K. Reishofer G. Ebner F. (2013). The function of the left angular gyrus in mental arithmetic: evidence from the associative confusion effect. Hum. Brain Mapp. 34, 1013–1024 10.1002/hbm.21489 22125269 PMC6870455

[B15] Griffin S. (2004). Building number sense with number worlds. Early Child. Res. Q. 19, 173–180 10.1016/j.ecresq.2004.01.01217407403

[B16] Howard-Jones P. A. Washbrook E. V. Meadows S. (2012). The timing of educational investment: a neuroscientific perspective. Dev. Cogn. Neurosci. 2S, S18–S29 10.1016/j.dcn.2011.11.002 22682906 PMC7110431

[B17] Hubbard E. M. Piazza M. Pinel P. Dehaene S. (2005). Interactions between number and space in parietal cortex. Nat. Rev. Neurosci. 6, 435–448 10.1038/nrn1684 15928716

[B18] Kroeger L. A. Brown R. D. O'Brien B. A. (2012). Connecting neuroscience, cognitive, and educational theories and research to practice: a review of mathematics intervention programs. Early Educ. Dev. 23, 37–58 10.1080/10409289.2012.617289

[B19] Kroger J. K. Nystrom L. E. Cohen J. D. Johnson-Laird P. N. (2008). Distinct neural substrates for deductive and mathematical processing. Brain Res. 1243, 86–103 10.1016/j.brainres.2008.07.128 18760263

[B20] Lee K. Lim Z. Y. Yeong S. H. Ng S. F. Venkatraman V. Chee M. W. (2007). Strategic differences in algebraic problem solving: neuroanatomical correlates. Brain Res. 1155, 163–171 10.1016/j.brainres.2007.04.040 17509541

[B22] National Council of Teachers of Mathematics. (2000). Principles and Standards for School Mathematics. Reston, VA: NCTM

[B23] OECD. (2010). The High Cost of Low Educational Performance: The Long-Run Economic Impact of Improving PISA Outcomes. Paris: OECD

[B24] Qin Y. Carter C. S. Silk E. M. Stenger V. A. Fissell K. Goode A. (2004). The change of the brain activation patterns as children learn algebra equation solving. Proc. Natl. Acad. Sci. U.S.A. 101, 5686–5691 10.1073/pnas.0401227101 15064407 PMC397478

[B21] RAND Mathematics Study Panel Loewenberg Ball D. (2003). Mathematical Proficiency for All Students: Toward a Strategic Research and Development Program in Mathematics Education. Santa Monica, CA: RAND

[B25] Räsänen P. Salminen J. Wilson A. J. Aunio P. Dehaene S. (2009). Computer-assisted intervention for children with low numeracy skills. Cogn. Dev. 24, 450–472 10.1016/j.cogdev.2009.09.003

[B26] Rivera S. M. Reiss A. L. Eckert M. A. Menon V. (2005). Developmental changes in mental arithmetic: evidence for increased functional specialization in the left inferior parietal cortex. Cereb. Cortex 15, 1779–1790 10.1093/cercor/bhi055 15716474

[B27] Royer J. M. (ed.). (2003). Mathematical Cognition. Portland, OR: Book News, Inc.

[B28] Starr A. Libertus M. E. Brannon E. M. (2013). Number sense in infancy predicts mathematical abilities in childhood. Proc. Natl. Acad. Sci. U.S.A. 110, 18116–18120 10.1073/pnas.1302751110 24145427 PMC3831472

[B29] Susac A. Bubic A. Kaponja J. Planinic M. Palmovic M. (2014). Eye movements reveal students' strategies in simple equation solving. Int. J. Sci. Math. Educ. 12, 1–23 10.1007/s10763-014-9514-4

[B30] Vecchiato G. Susac A. Margeti S. De Vico Fallani F. Maglione A. G. Supek S. (2013). High-resolution EEG analysis of power spectral density maps and coherence networks in a proportional reasoning task. Brain Topogr. 26, 303–314 10.1007/s10548-012-0259-5 23053602

[B31] Wilson A. J. Dehaene S. Pinel P. Revkin S. K. Cohen L. Cohen D. (2006). Principles underlying the design of “The Number Race,” an adaptive computer game for remediation of dyscalculia. Behav. Brain Funct. 30, 2–19 10.1186/1744-9081-2-19 16734905 PMC1550244

[B32] Zamarian L. Ischebeck A. Delazer M. (2009). Neuroscience of learning arithmetic —evidence from brain imaging studies. Neurosci. Biobehav. Rev. 33, 909–925 10.1016/j.neubiorev.2009.03.00 19428500

